# Metformin: An Emerging New Therapeutic Option for Targeting Cancer Stem Cells and Metastasis

**DOI:** 10.1155/2012/928127

**Published:** 2012-06-04

**Authors:** Ramandeep Rattan, Rouba Ali Fehmi, Adnan Munkarah

**Affiliations:** ^1^Division of Gynecology Oncology, Department of Women's Health Services, Henry Ford Health System, Detroit, MI 48202, USA; ^2^Departments of Pathology and Obstetrics and Gynecology, Wayne State University, Detroit, MI 48201, USA

## Abstract

Metastasis is an intricate process by which a small number of cancer cells from the primary tumor site undergo numerous alterations, which enables them to form secondary tumors at another and often multiple sites in the host. Transition of a cancer cell from epithelial to mesenchymal phenotype is thought to be the first step in the progression of metastasis. Recently, the recognition of cancer stem cells has added to the perplexity in understanding metastasis, as studies suggest cancer stem cells to be the originators of metastasis. All current and investigative drugs have been unable to prevent or reverse metastasis, as a result of which most metastatic cancers are incurable. A potential drug that can be considered is metformin, an oral hypoglycemic drug. In this review we discuss the potential of metformin in targeting both epithelial to mesenchymal transition and cancer stem cells in combating cancer metastases.

## 1. Introduction

Metastasis is the process by which cancer cells translocate from their primary site to distant organs and establish secondary tumors. This simplified definition does not do justice to the complex enigmatic phenomenon that still lacks clear understanding. It is mainly because of metastasis that most cancers become incurable and result in death. It is these metastatic cells that escape the effects of chemotherapy and result in poor patient outcomes. Thus, a deeper knowledge of the process is crucial to devise therapeutic interventions that will result in better outcomes and survival for patients with metastatic disease. 

Until recently, the initiation of metastasis was solely attributed to the process of epithelial to mesenchymal (EMT) transition where a differentiated tumor cell transforms into a more aggressive, motile, and resistant cell. A cell after undergoing EMT was thought to be able to break the confines of its parent tumor and travel via lymphatic-blood system to a new receptive environment, where it would establish a new tumor. A full understanding of the EMT process is lacking. Recently, research efforts from a number of sources led to the identification of cancer stem cells (CSCs) [1, 2]. Some of the data generated suggest that these CSC may be at the origin of cancer metastasis [3–5].

 All current and investigative drugs have been unable to prevent or reverse metastasis. A better understanding of the process is prerequisite to design successful drugs and strategies to manage the presently untreatable metastasis. One potential drug that can be considered for this aspect is metformin. Metformin is a well-established and widely prescribed oral hypoglycemic drug. Recently, the drug has gained attention for its potential anticancer effects [6–8]. In addition to its antitumorigenic effects, recent reports have demonstrated inhibition of EMT genes and specific targeting of stem cells by metformin, thus supporting its potential role in fighting cancer metastases.

In the present review, we briefly discuss the basis of metastasis with respect to EMT and CSC and summarize some of the early studies on metformin showing its potential to interfere both with the process of EMT and CSC.

## 2. EMT in Metastasis

Based on the canonical teaching, tumor metastasis is dependent on a small number of cancer cells in the primary tumor, which have the ability to undergo vast genetic changes. These changes will ultimately help those cells detach from the primary tumor location, implant at a separate site, and generate secondary tumors [9]. In order for the metastatic process to succeed, a cancer cell should be able to survive under attachment-free conditions, migrate and invade through the surrounding stroma, intravasate into the vascular system, endure and extravasate into an advantageous distant environment, adhere and proliferate [10]. Out of this complex multistep process, the initial steps of a cell gaining the ability of surviving adhesion-free, migrating, and invading the extracellular matrix are the most crucial.

EMT was first defined as a developmental cell program required in early embryogenesis [11]. In the carcinogenic process, EMT pertains to an epithelial cancer cell losing its epithelial makeup and acquiring mesenchymal characteristics that confer on it survival and migratory advantages [4, 11]. These include breakdown of the cell-cell junctions and cytoskeleton alterations, providing the cell with motility and invasiveness. This process works through a set of driving transcription factors that include Snail, Slug, Twist, ZEB1/2, and KLF-8 [3, 12–14]. The initial changes comprise the loss of expression of the epithelial marker E-Cadherin [15, 16] and gain in expression of Vimentin, N-Cadherin, and Fibronectin [17, 18], all associated with the mesenchymal phenotype. The question of how these cells acquire these genes is still at large. Investigators have shown that stimulation of pathways like Wnt [19], Notch [20, 21], hypoxia [21, 22], integrins [23], and PI3k-Akt [24] results in EMT-related changes.

 Identification of the essential role of microenvironment has also led to acknowledgement of its contribution in the metastatic process [25, 26]. Various studies have shown that tumor cells recruit and interact with the various components of the stroma to create a microenvironment conducive to pass and generate signals to initiate EMT [27]. Growth factors like TNF-*α* [28], TGF-*β* [29], FGF [30], EGF [31], PDGF [32], and even IGF [33] have been shown to induce EMT in myriad tumor cell lines. It has been suggested that the surface tumor cells that are in direct contact with the stromal components have the highest ability to proceed towards EMT.

A clear pathway as to what initiates or drives EMT is still unknown. Although EMT offers a plausible explanation of how cells become metastatic, a lot of questions still need to be answered. What decides which cells will undergo EMT? What is the first cue required by a cell to initiate EMT? What are the minimum alterations required to acquire metastatic phenotype? What is the process involved in the tumors originating from nonepithelial cells? Apart from the complexity of going through EMT, the cell has to successfully navigate through the blood vessels and reach a receptive seeding ground where it needs to turn off the EMT, undergo reverse mesenchymal to epithelial transition (MET) in order to adhere and start proliferating [15, 17]. Does the process of EMT also grant these additional characteristics? If not, how do cells acquire these required traits to complete the journey of metastasis?

Taking into account the intricate and enigmatic process leading to metastasis, it is astonishing that it occurs frequently in the clinical setting.

## 3. Cancer Stem Cells in Metastasis

Recent understanding of the heterogeneous makeup of the cancer cells in a tumor has revealed the presence of CSCs [1, 2]. The CSCs are distinguished by some major properties that include (i) self-renewing ability by asymmetric division (ii), ability to differentiate into diverse phenotypes, (iii) ability to initiate tumors from minute numbers, and (iv) high chemoresistance [1, 34–38]. The discovery of CSCs in cancer has caused a major shift in the understanding of cancer biology and is greatly affecting the investigation of new therapeutics as well. Presently, there is no clear explanation as to the origin of the CSCs. Many theories exist including that these cells have changed (mutated) from the hematopoietic or tissue specific or somatic stem cells, or that they have arisen due to the regression of a differentiated tumor cell. The first role of CSC in cancer was described in hematologic malignancies [39]. Lapidot et al. demonstrated that a small subset of leukemic cells characterized by CD34^+^CD38^−^ cell surface markers were able to generate tumors in SCID mice, reflecting disease similar to that seen in the original patient. Recently, many studies have elegantly demonstrated the self-renewal, resilient, and tumorigenic properties of CSCs in tumors like breast [2], head and neck carcinoma [40], brain tumor [41, 42], colon cancer [43, 44], melanoma [45, 46], liver [47], prostate [48], and ovarian [49]. CSCs isolated from different tumors seem to display a variety cell surface markers: colon and brain CSC have been isolated using CD133^+^, prostate CSC with CD44^+^a2b1^+^ breast with CD44^+^CD24^−^, ovarian with ALDH^+^, and so forth. In addition, these cells do coexpress pluripotent embryonic cell markers like c-myc, Nanog, Sox2, Klf-4, Oct 4 and lin 28 [50–52]. The inherent capability to initiate a tumor from a very small number of CSCs (counts of hundreds) in immuno-compromised mice has been shown by various researchers, a property not shared by the regular cancer cells. Based on these findings, the aggressiveness and recurrence of tumors are now being attributed to the minute population of CSCs residing within the cancer cells. Although miniscule in number, they have been anointed by many to be solely responsible for driving and maintaining various tumors.

Thus, could it also be possible that it is these CSCs that are also responsible for metastasis? If we observe the aspects exhibited by a CSC, like initiating cancer from limited number of cells, longevity, enhanced resistance to the apoptotic process, and remarkable motility, they appear to be the ideal cells to cause metastasis. Supporting this is also the increasing observations that cells that undergo EMT have a CSC-like state [3–5, 53, 54], indicating that having properties germane to a stem cell are required for successful metastasis. Experts in the field have debated whether the CSCs acquire/reexpress EMT traits when they are ready to metastasize or the differentiated tumor cells revert back to a more plastic stage based on their gain of EMT signature. Our speculation is that both types of prospective-metastatic cells exist within the tumor: the CSCs with inherent metastatic potential and the acquired-EMT-turned-CSC tumor cells.

This knowledge gives arise to several other questions: which cell actually initiates the process of metastasis? What are the cues that would compel the cell (either CSC or EMT tumor cell) to initiate metastasis? Is it the CSC/tumor cell that first initiates the cross-talk with the stromal microenvironment or is it the stroma that provides the initial signals to the cell to change? These questions further confuse the already clouded biology of metastasis. One hypothesis in this regard suggests that the microenvironment around the CSC specifically initiates the signals. The small population of CSC seems to reside in a discrete milieu called the “stem cell niche” [55], an ideal environment that maintains self-renewing asymmetric dividing capability and provides protection against stress and toxic insults [56]. Most CSC niches are found adjoining blood vessels [46, 57], making it more favorable for cells to intravasate and disseminate. It has also been postulated that this type of microenvironment induces the differentiated cancer cells to undergo EMT and induce a CSC-like phenotype [58], which eventually results in metastasizing of tumor cells.

This emerging concept of the CSCs being responsible for metastasis along with their high resistance to conventional chemotherapy suggests them to be the ultimate foe in combat against cancer. Thus, it is not surprising that therapies that would specifically abolish these CSC are being actively investigated. In this regard, we discuss the potential role of metformin in targeting stem cells to control tumor progression and more importantly limit metastasis.

## 4. Metformin in Cancer

Metformin belongs to a class of compounds called biguanines that were first isolated from the plant *Galega officinalis* (French lilac or goat's rue) known for its medicinal value [59]. It was first described in 1922 by Werner and Well, and its glucose lowering action was first documented in 1929 by Slotta and Tschesche. But it was only in the late 1950s that metformin was established as a glucose-lowering drug and became available for human consumption in UK. Slowly, it gained worldwide interest and was approved by the FDA in 1995 in the US [59, 60]. Today, it is the most widely prescribed antidiabetic drug for type 2 diabetes in the world [60]. Metformin's beneficial effects in diabetic patients has been shown to be largely through repression of hepatic gluconeogenesis, which reduces the glucose levels. In addition, it also increases insulin sensitivity and glucose uptake. The mechanism behind these actions is largely believed to be the inhibition of mitochondrial oxidative phosphorylation leading to an ATP/AMP imbalance, which results in activation of the LKB1-AMPK pathway [61]. Activation of AMPK, an enzyme that is the central regulator of metabolic pathways [62], has been credited with the glucose lowering effects seen with metformin.

In the past decade, metformin has gained wide attention for its anticancer properties. Numerous studies have shown that *in vitro* treatment with metformin inhibited the growth of myriad cancer cell lines including breast [6], glioma [7], renal cell [63], pancreatic [64], colon [65], ovarian [8], endometrial [8, 66], prostate [67], and lung [68]. Diverse *in vivo* models have also been used to demonstrate the antitumor abilities of metformin. One of the first reports was by Schneider et al. [69] where they showed metformin to inhibit carcinogen induced pancreatic cancer in high-fat diet fed hamsters. Another early study done in breast cancer mouse model showed that metformin treatment significantly decreased the tumor burden and accumulation of mammary adenocarcinomas accompanied by increase in the life span of HER-2/*neu* transgenic mice [70]. Lately, many other xenograft and genetic *in vivo* models have been used to describe the antigrowth effect of metformin in various tumor types [70–73]. Taking a step further, studies have also shown the advantages of combining metformin with standard therapeutics like cisplatin [71], taxol [74], and doxorubicin [75].

**Figure 1:**
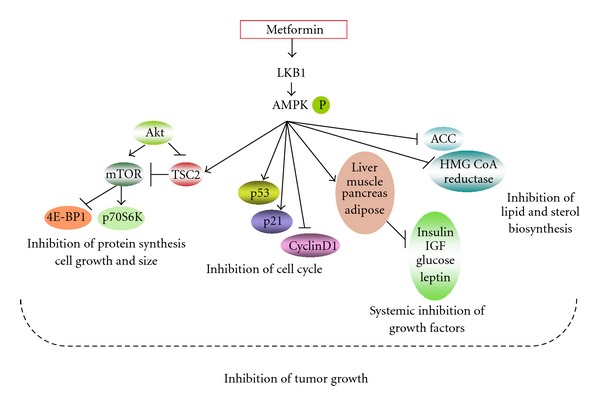
Downstream effects of AMPK activation by metformin resulting in inhibition of tumor growth. Activation of AMPK by metformin results in myriad effects that include (i) inhibition of mTOR, resulting in inhibition of protein synthesis and cell growth; (ii) activation of p53 and p21 along with inhibition of cyclins, resulting in cell cycle arrest; (iii) inhibition of lipid and sterol biosynthetic pathways; (iv) having a systemic effect on vital organs involved in glucose balance that results in reduced levels of growth factors like insulin, IGF, and leptin.

The main mechanism of tumor growth inhibition by metformin has been attributed to activation of AMPK leading to various downstream effects that work together to restrain tumor growth.  Figure 1 briefly enumerates the various downstream effects of metformin-mediated AMPK activation that have been inspected as being the mechanism(s) by which tumor growth is inhibited. One of the established and most investigated is the inhibition of the mTOR pathway [76]. Other modulators of the cancer inhibitory effects of metformin via AMPK activation include cyclin D1, p21, p27, Akt and p53 [8, 67]. AMPK has also been emerging as a chief player in autophagy, a phenomenon shown to be involved in tumor regulation [62, 77–81]. In addition, AMPK is required in some tumors for the transcriptional activity of HIF-1, a transcription factor that is crucial for adaptation of tumors to hypoxic environment [82]. Metformin-mediated activation of LKB1 could also result in the activation of other AMPK-related kinases [83]. These AMPK-related kinases may be performing functions similar to AMPK as shown by NUAK2 studies [84, 85].

Studies have also suggested that some of the effects of metformin are due to lowering of insulin levels [86], which acts as a tumor-promoting factor. Metformin intake has also been shown to create conditions of calorie restriction in the host, an ameliorating factor for tumor growth [87]. Some recently published data also shows metformin to be acting through AMPK-independent mechanisms [8, 88, 89].

A strong body of epidemiologic evidence that supports the anticancer benefits of metformin comes from data collected in diabetic patients. One of the first studies published by Evans et al. reported an association between metformin intake and lower cancer incidence [90]. Another large 1000-person retrospective study showed a significant decrease in cancer mortality for patients on metformin [91]. Recently, this was supported by an analysis of the ZODIAC trial data, where metformin takers were found to have a lower cancer-related mortality [92]. In a single center retrospective study, long-term intake of metformin (>36 months) was also associated with significant decrease in cancer risk [93]. Libby et al. reported that metformin users had lower incidence of cancer and delayed cancer occurrence time compared to nonusers [94]. Many other studies have also shown similar significant correlations in pancreatic [95] and breast cancer [96]. However, few other investigators failed to confirm this significant trend of lower frequency of cancer in diabetic patients [97–99].

A recent study of diabetic patients with colorectal cancer showed decreased cancer-related mortality in patients on metformin [100]. Studies have also inspected the association between metformin use and outcomes after chemotherapy in different cancers types. Jiralerspong et al. found that diabetic breast cancer patients receiving metformin along with taxane chemotherapy had a significant improvement in pathologic complete remission rate [101]. On the other hand, another study showed no additional benefit of metformin when combined with neoadjuvant chemotherapy [102]. It is important to realize that all of these reports present number of limitations. Most of the patients included have type 2 diabetes mellitus but may also be taking other medication to manage their diabetes or other illnesses. This confounds the data as to the true effects of metformin intake by itself.

The very interesting results of these retrospective studies have prompted researchers to plan clinical trials that may help further elucidate the antineoplastic effect of metformin. While many of them focus on breast cancer patients, few include other tumors like prostate, endometrial, kidney, pancreatic, and lung. A complete list as of October 2011 obtained from http://www.clinicaltrials.gov/ is compiled in Table 1. A couple of initial reports from these pilot studies show promising outcomes. In one study, nondiabetic breast cancer patients were randomized to metformin or no treatment for 2 weeks prior to undergoing breast surgery. Tumors of patients on metformin showed a significant reduction in the proliferation marker, Ki-67, suggesting that metformin may slow tumor cells proliferation. [103]. Another randomized pilot study showed that the intake of metformin for one month reduced the number of rectal aberrant crypt foci, an endoscopic surrogate marker of colorectal cancer [104]. The results of other clinical trials are eagerly awaited and would hopefully provide further insights on the anticancer benefits of metformin.

## 5. Metformin against Metastasis

Two theories have been proposed to explain metastasis: EMT and CSC. Regardless which mechanism prevails, reports showing metformin mitigating both EMT and CSC support the potential use of metformin in preventing metastasis. Few reports investigate the action of metformin against metastasis or stem cells.

### 5.1. Metformin Inhibits EMT

One of the first studies on this topic was by Beckner et al. where they reported metformin to inhibit *in vitro* migration of glycolytic glioma cells [105]. Hwang et al. detailed the antimetastatic ability of metformin in an *in vitro* study with fibrosarcoma cells [106]. They demonstrated that metformin inhibited *in vitro* migration and invasion of fibrosarcoma cells by a CamK-dependent pathway. Phoenix et al. studied the effect of high calorie diet on aggressiveness and metastasis of triple negative breast cancer in a syngeneic *in vivo* model and its amelioration with calorie restriction and metformin. In mice that were fed a high-energy diet, metformin was able to limit the growth of breast primary tumor but was unable to restrict metastatic nodules in the lung [107]. On the other hand, a report from Vazquez-Martin et al. [108] reported metformin to repress the metastasis-associated protein CD24, in a triple negative breast cancer cell line. Another interesting study was done in endometrial adenocarcinoma cells [109], using sera from polycystic ovarian syndrome (PCOS) patients before and after 6-month metformin therapy. PCOS is a major risk factor for endometrial cancer and is associated with metabolic syndrome and inflammation [110]. The investigators reported that sera from PCOS patients caused increased migration in endometrial cancer cells, while sera from PCOS metformin-treated patients caused significant inhibition of migration. This was also associated with inhibition in NF*κ*B, MMP-9, and MMP-2 activities and decreased Akt and Erk1/2 phosphorylation. We have recently shown that metformin reduced the number and size of metastatic lung nodules in a xenograft model of ovarian cancer [71]. Activation of the mTOR-S6K pathway has been associated with the epithelial-mesenchymal transition phenotype. Overexpression of p70S6K has been correlated with underexpression of E-cadherin and overexpression of N-cadherin and vimentin, shown to be mediated by Snail [111]. Since treatment with metformin has been shown to inhibit the mTOR-S6K pathway, this could also be one possible mechanism by which metformin inhibits EMT and metastasis. In a recent clinical study, patients with triple negative breast cancer who were on metformin had a lower risk of distant metastasis compared to women who were not on the drug [102].

### 5.2. Metformin Targets Stem Cells

The first report defining metformin's specific action against stem cells was published by Hirsch et al. The authors demonstrated that breast cancer stem cells, characterized by CD44^high^ CD24^low^ phenotype, are susceptible to metformin at low doses that do not affect the tumor cells. They showed *in vitro* and *in vivo* that metformin can eliminate CSCs and virtually eradicates breast tumors in mice when given along with doxorubicin [75]. Vaquez-Martin et al. presented evidence that CD44^+^CD24^−^ CSCs in HER2-positive breast cancer cells lines, that are resistant to trastuzumab, have selective sensitivity to low doses of metformin. They further showed metformin to act synergistically with trastuzumab to repress proliferation and survival of CSC in HER2-positive breast cancer cell lines [112]. The same group also demonstrated that metformin delays the EMT-driven acquisition of stem cell phenotype and the formation of self-renewing mammospheres that may represent “micro-tumors.” This occurred by inhibition of key EMT transcription factors like ZEB1, TWIST, Slug, and TGF-*β*. TGF-*β* has been found to be involved in closely regulating the process of EMT as well as appearance of CSC-like cells, [3, 113, 114], particularly in breast cancer cells [115, 116]. Metformin was also able to inhibit the progression of TGF-*β*-induced EMT changes by retaining the expression of E-cadherin and preventing concurrent appearance of vimentin expression, two events that occur when an epithelial cancer cell converts into a mesenchymal cell [116].

There has been some suggestions that cancer stem cells may be regulated by the mitochondria and metabolic reprogramming [117, 118]. Certain metabolites like high-energy lactanes and ketones promote the “stemness” of cancer cells by upregulating stemness-associated genes as well as genes found in embryonic stem cells. These gene signatures, induced ketone and lactate, were correlated with poor patient survival. It was speculated that this was due to fueling of the tumors by pushing them towards oxidative phosphorylation [119]. With this respect, metformin is known to interfere with the mitochondrial process and can attenuate the metabolic changes enabled by ketone/lactate metabolites (or other metabolites).

**Figure 2:**
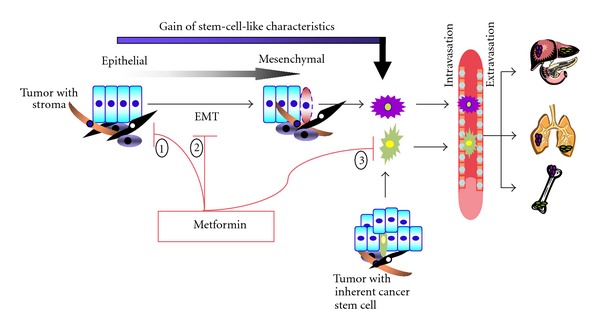
Metformin can inhibit appearance of metastasis by (1) limiting growth of the primary tumor; (2) inhibiting EMT; (3) eradicating cancer stem cells.

 These initial studies demonstrating the effects of metformin against CSCs look promising and need to be expanded to gain further insight into the specific action and the mechanism involved. Overall, metformin seems to be a viable therapeutic choice to target metastasis as it seems to affect both the EMT and cancer stem cells (Figure 2), both believed to be focal points for metastasis.

## 6. Concluding Remarks

Our present knowledge is far from a complete understanding of the complex and multifaceted process of metastasis. The discovery of CSC population offers an explanation for some of the unique behaviors seen in cancer. The emerging studies showing eradication of animal tumors by double targeting of cancer cells and CSCs provide significant hope for the future.

The emergence of metformin as a potential anticancer and cancer-preventive therapeutic tool is exciting. With the added benefits of being readily available, economical, and easily tolerated with good safety profile, it can be effortlessly transitioned from bench to bedside for cancer therapy.

## Figures and Tables

**Table 1:** 

Identification & Title	Primary Goals	Status
**NCT01340300**		
Randomized Phase II study of exercise and metformin in colorectal cancer survivors	Randomized study will compare interventions with exercise and/or metformin, with a control arm. Insulin levels and other blood markers will be estimated which may indicate ecurrences	Not yet recruiting
**NCT01266486**		
A Phase 2 single arm study to examine the effects of metformin on cancer metabolism in patients with early stage breast cancer receiving neoadjuvant chemotherapy	Pre-surgery metformin will be given to patients for 3 weeks. Lipid metabolism of the tumor will be studied. Patients will have option of taking metformin along with neoadjuvant chemotherapy. Metformin induced effects in phosphorylation of S6K, 4E-BP-1 and AMPK will be estimated by IHC	Recruiting
**NCT01302002**		
A Phase 0 study regarding the biological effects of use of metformin in early breast cancer patients pre-surgery	After 3 weeks of metformin intake, in situ effects of metformin will be determined in women with operable stage I or II breast cancer. Pre and post tissues will be compared for proliferation (Ki67), apoptosis (TUNEL) and fosforilate AKT	Recruiting
**NCT00897884**		
Interventional study of clinical and biologic effects of metformin in early stage breast cancer	After 2-3 weeks of metformin intake, pre- and post-operative biopsy samples will be compared for proliferation	Recruiting
**NCT01210911**		
A Phase II, randomized, placebo controlled study to evaluate the efficacy of the combination of gemcitabine, erlotinib and metformin in patients with locally advanced and metastatic pancreatic cancer	Survival after 6 months of combinational therapy will be determined	Recruiting
**NCT01440127**		
Phase I randomized clinical trial evaluating the impact of pretreatment with metformin on colorectal cancer stem cells and related pharmacodynamic markers	Pateints will randomly receive metformin pre-procedure for approximately 1 week. Cancer stem cells will be isolated from blood. Glucose will be measured	Recruiting
**NCT01205672**		
Interventional non-randomized evaluation of the molecular effects of metformin on the endometrium in patients with endometrial cancer	30 days before surgery patients will be given metformin. Molecular effects of metformin will be measured by changes in insulin/glucose metabolism on the mTOR signaling in endometrium of women with endometrial cancer and high body mass index	Recruiting
**NCT00984490**		
Interventional pre-surgical trial of metformin in patients with operable breast cancer	After 1–3 weeks of metformin intake, pre- and post-operative biopsy samples will be compared for proliferation ( Ki67) in women with stage I or stage II breast cancer that can be removed by surgery	Recruiting
**NCT01310231**		
A randomized Phase II, double blind, trial of standard chemotherapy with metformin (vs. placebo) in women with metastatic breast cancer receiving first or second line chemotherapy with anthracycline, taxane, platinum or capecitabine based regimens	Metformin will be given along with standard chemotherapy. Progression free survival will be assessed up to 3 years	Recruiting
**NCT01433913**		
Phase II study of metformin in a pre-prostatectomy prostate cancer cohort	Metformin will be given for 4 to 12 weeks before surgical removal of the prostate gland. Levels of metformin will be detected in prostate tissue. Physiological and cellular abnormalities in prostate tissue removed at surgery will be measured	Not yet recruiting
**NCT01333852**		
Randomized Phase II study of paclitaxel plus metformin or placebo for the treatment of platinum-refractory, recurrent or metastatic head and neck neoplasms	Various combinations will be given to patients and Disease Progression-free survival at 12 weeks and 6 months will be recorded	Recruiting
**NCT00659568**		
A Phase I study of temsirolimus in combination with metformin in advanced solid tumours	Maximum tolerated dose and recommended phase II dose of metformin along with temsirolimus will be estimated. Antitumor activity, including tumor response rate and time to progression will be recorded	Completed
**NCT01215032**		
Prospective study of metformin in castration-resistant prostate cancer	Metformin will be given along with androgen deprivation therapy in a 2-year study. PSA (prostate specific antigen) response will be monitored	Recruiting
**NCT01101438**		
A Phase III randomized trial of metformin versus placebo on recurrence and survival in early stage breast cancer	Patients will intake metformin for 5 years. Invasive disease-free survival and Overall survival will be recorded	Recruiting
**NCT00881725**		
A Phase II, open label assessment of neoadjuvant intervention with metformin against tumour expression of signaling prostate cancer	Patients will take metformin for 4–12 weeks prior to Radical Prostatectomy. Difference in P-AKT staining and other parameters will be measured under pre- and post-surgery conditions	Active, not recruiting
**NCT01087983**		
Phase 1 trial of lapatinib in combination with (1) sirolimus or (2) metformin in advanced cancer	Maximum Tolerated Dose (MTD) of Lapatinib with the combinations will be calculated	Recruiting
**NCT01243385**		
Metformin in castration resistant prostate cancer. A multicenter Phase II trial	Safety of giving metformin as first-line therapy in treating patients with locally advanced or metastatic prostate cancer will be assesed. Progression-free survival (PFS) at 12 weeks and at later time points with continuation of therapy will be recorded	Recruiting
**NCT01341886**		
Effect of metformin on decrement in levothyroxin dose required for thyroid stimulating hormone (TSH) suppression in patients with differentiated thyroid cancer	Metformin will be given as an additional drug to levothyroxin in order to decrease levothyroxine dosage by 30%. Metformin's effect in inducing TSH suppression without change in T3 and T4 concentration will be estimated	Completed
**NCT01430351**		
A Phase I lead-in to a 2 × 2 × 2 factorial rrial of dose dense temozolomide (TMZ), memantine (MEMTN), mefloquine (MFLOQ), and metformin as post-radiation adjuvant therapy of glioblastoma multiforme	The study will determine the safety and tolerability of TMZ in combination with Metformin and/or (MFLOQ) and/or MEMTN in patients receiving adjuvant therapy after completing external beam radiotherapy for newly diagnosed glioblastoma multiforme. Median progression free survival at 6, 12, and 18 months will be measured	Recruiting
**NCT01167738**		
A randomized Phase II study of chemotherapy and/or not metformin in metastatic pancreatic cancer	Studying giving cisplatin, epirubicin, capecitabine, and gemcitabine together with metformin to see how well it works compared to chemotherapy alone in treating patients with metastatic pancreatic cancer. Progression-free survival at 6 months and overall survival will be estimated	Recruiting
**NCT01447927**		
A Phase II trial of metformin in preventing esophageal cancer in patients with barrett esophagus	Effect of metformin intake for 2–12 weeks in preventing esophageal cancer in patients with Barrett esophagus will be observed. Percent change in the mean pS6K1 immunostaining will be taken as marker	Not yet recruiting
**NCT01312467**		
A Phase IIA trial of metformin for colorectal cancer risk reduction among patients with a history of colorectal adenomas and elevated body mass index	To determine if a 12-week intervention of oral metformin treatment among obese patients with a history of colorectal adenomas results in at least a 35% decrease in colorectal mucosa. Activated pS6serine235 from baseline as assessed via immunostaining in pre and post biopsies	Recruiting
**NCT00909506**		
A Phase II trial of efficacy and safety of adjuvant metformin for operable breast cancer patients	Metformin will be given to patients of operable breast cancer patients with overweight or pre-DM for 24 weeks, to test the efficacy and safety of adjuvant metformin and weight loss	Recruiting
**NCT00930579**		
A Phase II pre-surgical intervention study for evaluating the effect of metformin on breast cancer proliferation	Effects of metformin on AMPK/mTOR signaling pathway and insulin levels will be measured after 2 weeks on metformin	Recruiting
**NCT01442870**		
A Phase I prospective evaluation of clinical safety of combining metformin with anticancer chemotherapy	Cytologically documented cancer patients will be given metformin for 3 weeks to determine whether metformin can be safely added to a chemotherapy regimen that is previously well tolerated. The rate of dose limiting toxicities will be compared	Recruiting
**NCT01324180**		
A Phase I window, dose escalating and safety trial of metformin in combination with induction chemotherapy in relapsed refractory acute lymphoblastic leukemia: metformin with induction chemotherapy of vincristine, dexamethasone, doxorubicin, and PEG-asparaginase (VPLD)	Clinical and biological effects of metformin in combination with standard systemic chemotherapy in relapsed ALL patients that have a dismal outcome will be estimated. A dose escalation study to find the Maximum Tolerated Dose (MTD) of metformin in conjunction with ALL therapy. Complete Remission will be taken as end point	Recruiting
